# Detection and genetic characteristics of porcine circovirus 3 based on oral fluids from asymptomatic pigs in central China

**DOI:** 10.1186/s12917-019-1952-3

**Published:** 2019-06-13

**Authors:** Zhenhua Guo, Xiang Li, Ruiguang Deng, Gaiping Zhang

**Affiliations:** 1 Key Laboratory of Animal Immunology of the Ministry of Agriculture, Henan Provincial Key Laboratory of Animal Immunology, Henan Academy of Agricultural Sciences, Zhengzhou, People’s Republic of China; 2 College of Animal Science and Veterinary Medicine, Henan Agricultural University, Zhengzhou, 450002 People’s Republic of China; 3 Jiangsu Co-innovation Center for Prevention and Control of Important Animal Infectious Diseases and Zoonoses, Yangzhou, People’s Republic of China; 4 Department of Veterinary Medicine, Yunnan Agricultural University, Kunming, People’s Republic of China

**Keywords:** PCV3, Epidemiology, Oral fluids, Phylogenetic analysis

## Abstract

**Background:**

Porcine circovirus 3 (PCV3) is an emerging etiological agent to the swine industry. However, its circulating status and genetic characteristics were still unclear in Henan, central China. Here, 318 porcine oral fluid specimens were collected from asymptomatic pigs in five farms and tested by PCR .

**Results:**

The results showed that the positive rate of PCV3 was 12.3% (39/318) for the total samples, and 15.06% (25/166) in the stall-based samples, 9.21% (14/152) in the pen-based samples. Of the PCV3-positive samples, 41.0% were also positive for porcine circovirus 2 (PCV2). Nucleotide sequence comparison indicated that the 10 complete genomes and 34 capsid (*cap*) genes in this study shared 98.7–99.9% and 98–100% pairwise identities to each other, respectively. According to phylogenetic analysis and sequence alignment of *cap* gene, all the isolated sequences were clustered into 3 clades, including subgroup 1 (21/39, 61.8%), subgroup 2 (5/39, 14.7%) and subgroup 3 (8/39, 23.5%). Similar to previous reports, four amino acids (V24A, K27R, S77 T and I150L) in cap protein were identified as a conserved subgroup specific molecular marker.

**Conclusion:**

Our research provided new insights into the epidemiology surveillance and genetic characteristics of PCV3 in China.

**Electronic supplementary material:**

The online version of this article (10.1186/s12917-019-1952-3) contains supplementary material, which is available to authorized users.

## Background

Circoviridae family have covalently closed, circular, single-stranded DNA (ssDNA) genomes and are classified into two genera: *Circovirus* and *Cyclovirus* [1]*.* Two members of the circovirus genus, PCV type 1 (PCV1) and PCV type 2 (PCV2), have been recognized as infectious agents to pigs. PCV1 was first detected as a contaminant of PK-15 cell cultures and has not been associated with clinical disease [2]. However, PCV2 is a ubiquitous economically pathogen to the swine industry. PCV2 infection leads to a diverse range of clinical diseases collectively termed PCV2-associated disease (PCVAD), which includes post-weaning multi-systemic wasting syndrome (PMWS), porcine dermatitis and nephropathy syndrome (PDNS), interstitial pneumonia, enteric disease and reproductive failure [3].

Recently, a novel porcine circovirus, designated as porcine circovirus 3 (PCV3), was first identified from a case of porcine dermatitis and nephropathy syndrome (PDNS) and reproductive failure in USA in 2015 [4, 5]. The genome size of PCV3 is ~ 2000 nucleotides (nt), which is considerably longer than PCV1 (~ 1760 nt) and PCV2 (~ 1780 nt), respectively [6]. Currently, PCV3 has been reported in several countries such as China, South Korea, Thailand, Poland, Italy, and Brazil, accompanying with widely geographical distribution [7–12]. Many studies have shown that PCV3 was probably a potential pathogen which was related with several clinical diseases, including PDNS, reproductive disorders and multi-systemic inflammation [4, 5, 13] .

In China, PCV3 has been confirmed to be present in more than 20 provinces and regions [8, 14, 15]. However, the prevalence of PCV3 in pig farms (especially in asymptomatic herds) is still not well established and the genetic information of PCV3 is still poor in central China. The aim of this study was to determine the infection status and genetic characteristics of PCV3 in asymptomatic pigs using pen-based and stall-based oral fluid samples in Henan, central China.

## Results

### The detection result of PCV3 based on oral fluids

Of the total 318 oral fluid samples, 12.3% (39/318) were positive for PCV3 DNA, and 15.06% (25/166) in the stall-based samples from sows, 9.21% (14/152) in the pen-based samples from commercial pigs. For individual farm, the detection rates of PCV3 were ranged from 4.0%~ 20.0% (Table 1). Furthermore, we also addressed the co-infection status of PCV2 with PCV3. Of the 39 PCV3-positive samples, 16 samples (~ 41.0%) were also positive for PCV2. Besides, our data showed that there was a higher infection rate of PCV3 in sows (15.06%) compared with that in commercial pigs (9.21%), and the prevalence of PCV3 is common in pig farms, even if there are no obvious clinical signs.

**Table 1:** Sample information and the detection result of PCV3

Farm	Herd size (Sows)	Sample size		Detection rate of PCV3		
		Sows (Stall-based)	Commercial pigs (Pen-based)	Sows	Commercial pigs	Total
A	600	33	27	24.24% (8/33)	14.81% (4/27)	20.0% (12/60)
B	900	35	28	11.43% (4/35)	7.14% (2/28)	9.5% (6/63)
C	300	20	34	20% (4/20)	14.71% (5/34)	16.7% (9/54)
D	400	36	30	16.67% (6/36)	10% (3/30)	13.6% (9/66)
E	1000	42	33	7.14% (3/42)	0% (0/33)	4.0% (3/75)
Total	3200	166	152	15.06% (25/166)	9.21% (14/152)	12.3% (39/318)

### Sequence analysis

To further analyze the sequences of PCV3 variants, the nucleotide sequences of 10 complete (2000 nts) genomes (GenBank accession numbers: MH184533-MH184542) and 34 complete *cap* (645 nts) genes (GenBank accession numbers: MH184533-MH184566) were sequenced and analyzed (Additional file 1: Table S1). The sequence identities of whole genome and *cap* gene in this study were 98.7%~ 99.9% (nt) and 98%~ 100% (nt) (96.7%~ 100%, aa), respectively. Besides, early isolates or representative strains of PCV1, PCV2 and PCV3 were chose for further analysis. These new strains showed a high identity with the American PCV3 reference strains (GenBank ID: KX778720, KX966193) and the similarity was 98.7%~ 99.5% (nt) for complete genome and 98.0%~ 99.8% (nt) (98.1%~ 100%, aa) for *cap* gene, respectively. However, they only shared 45.3%~ 45.5% genomic nucleotide identity with PCV1 reference strains (GenBank ID: KJ408798, DQ650650) and 46.0%~ 46.8% identity with PCV2 reference strains (GenBank ID: EU148503, AF055394, FJ870968), respectively (Table 2).

**Table 2:** The homological analysis between different strains

Isolated strains in this study	Each other	PCV3 reference strain (KX778720, KX966193)	PCV1 reference strain (KJ408798, DQ650650)	PCV2 reference strain (FJ870968, AF055394, EU148503)
Complete genome (nt)	98.7%~ 99.9%	98.7%~ 99.5%	45.3%~ 45.5%	46.0%~ 46.8%
Cap gene (nt/aa)	98.0%~ 100%/	98.0%~ 99.8%/	44.0%~ 44.8%/	43.7%~ 45.1%/
	96.7%~ 100%	98.1%~ 100%	23.0%~ 25.4%	25.9%~ 26.4%

### Phylogenetic analysis and sequence alignment

**Fig. 1:**
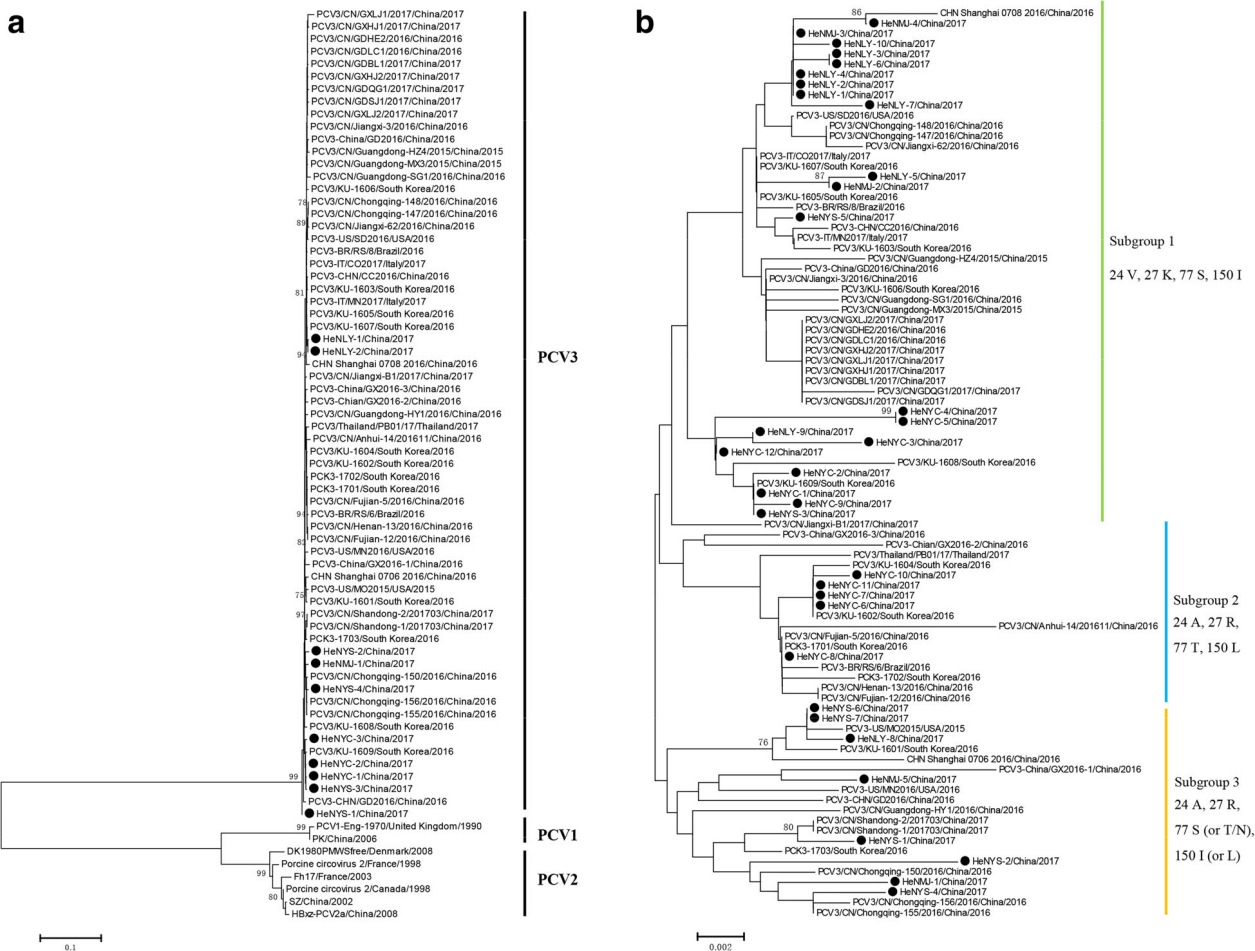
Phylogenetic analysis based on the complete genome and the cap gene. **a** Phylogenetic tree of the complete genome of different porcine circoviruses. All PCV3 strains cluster together independently with PCV1 and PCV2 species. **b** Phylogenetic tree and amino acids alignment based on the cap gene of 89 viruses. All the PCV3 strains clustered three subgroups, subgroup 1, subgroup 2 and subgroup 3. The special amino acid patterns for each subgroup were also showed. The black solid circles (●) represent the PCV3 viruses investigated in this study. The phylogenetic tree was constructed using the neighbour-joining method in MEGA 6.0 software with a bootstrap test of 1000 replicates. Scale bar indicates nucleotide substitutions per site

**Fig. 2:**
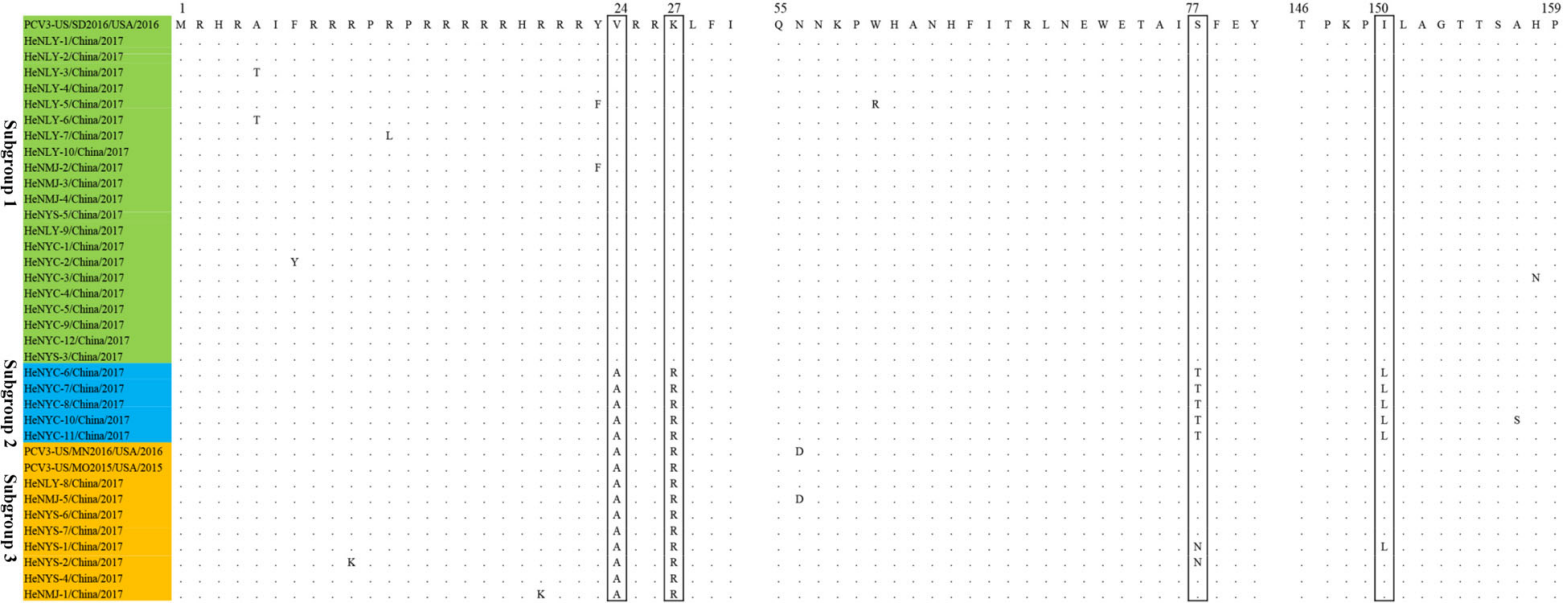
The sequence alignment based on amino acids of capsid protein. 34 PCV3 strains isolated in this study and 3 reference strains were aligned by clustal W method in MEGA6.0 software. All the isolates were cluster into three subgroups, subgroup 1 (green), subgroup 2 (blue), subgroup 3 (yellow). The potential genetic marker were showed in the black rectangles

To investigate the evolutionary relationship of PCV3 in China, a total of 73 complete genomes (10 obtained from this study and 63 downloaded from GenBank) and 89 cap gene sequences (34 sequences from this study and 55 sequences from GenBank) were analyzed using the neighbor-joining method in MEGA6.0 software. Based on the complete genome sequences, all the PCV3 were clustered into a same but separate evolutionary branch, which were distantly related to PCV1 and PCV2 species (Fig. 1a). The phylogenetic tree based on the full-length *cap* gene showed that all the 89 PCV3 strains could be divided into three subgroups-subgroup 1, subgroup 2 and subgroup 3 (Fig. 1b). Among the 34 PCV3 sequences obtained presently, 61.8% (21/34) sequences were clustered into subgroup 1, 14.7% (5/34) belonged to subgroup 2 and 23.5% (8/34) belonged to subgroup 3. Sequence alignment based on the cap protein showed that the different subgroups possess different amino acid patterns at position 24th, 27th, 77th and 150th (Figs. 1b and 2). Concisely, subgroup 1 contains a combination of 24 V, 27 K, 77S and 150I. Subgroup 2 contains 24A, 27R, 77 T and 150 L. While subgroup 3 contains 24A, 27R, 77S (or T/N) and 150I (or N/L). Further investigations need to be done to determine whether these four amino acids variation were related to pathogenicity or antigenicity of different PCV3 subgroups.

## Discussion

PCV3 was first identified as a potential etiologic agent to swine in the America in 2015 [4, 5]. Since then, several countries (China, South Korea, Thailand, Poland, Italy, Brazil, etc.) have reported the prevalence of PCV3 [7–12, 14]. The retrospective studies showed that the PCV3 were detected in pigs as early as in 1993 in Sweden and 1996 in China and Spain, respectively. It suggests that PCV3 has been circulating in pigs more than 20 years [16, 17].

Based on the detection using anti-PCV3 capsid antibodies, Rachel Palinski et al. reported a 55% positive rate of serum samples in USA, Junhua Deng et al. showed a 29.63%~ 61.67% positive rate based on serum samples collected from 15 provinces/regions in China. These studies indicated that PCV3 is commonly circulated in the pig farms with a high infection rate. In our study, The PCV3 positive rates range from 4 to 20% for individual pig farm and the sows showed a higher detection rate of PCV3 (15.06%) than finishing pigs (9.21%). The total detection rate of PCV3 (12.3%) was lower than that in previous reports in China, a positive rate of 34.7 and 21.9%~ 31.1% [8, 15], respectively. This is probably caused by the discrepancy of sample sources and types. For most previous studies, the samples were serums and tissues (lung, lymph node, tonsil, kidney, etc.) collected from diseased pigs, while the samples we used were oral fluids collected from the asymptomatic pig farms. Furthermore, we confirmed that the co-infection rate with PCV2 was 41.0%, which was similar with previous studies.

Homological analysis indicated that the genome of PCV3 isolates had a high identity (98.7%~ 99.9%) with each other. Based on the phylogenetic analysis and sequence alignment of cap gene sequences, all the isolates could be classified into subgroup 1, subgroup 2 and subgroup 3. Besides, similar to previous researches, four amino acid variations (V24A, K27R, S77 T and I150L) in cap protein could be used as a molecular marker for different PCV3 clade divisions. In fact, several studies have discussed the genotyping of PCV3 strains. Robert et al. (2018) thought there are at least two separate groups of PCV3 strains and they also provided the amino acid marker positions in ORF1 and 2 of PCV3 strains [18]. X. Ku et al. (2016) considered there are two clusters of PCV3 based on the phylogenetic analysis of partial capsid gene [8]. X. Fu et al. (2017) divided PCV3 into three clades (PCV3a, PCV3b and PCV3c) based on two amino acid mutations (A24V and R27K) on the cap protein [15]. As a newly found circovirus species, there was still no standardized nomenclature for PCV3 genotype definition. Along with the increase of PCV3 sequence information, the researchers may be confused by different classifications of PCV3. Therefore, it is necessary to determine the definite nomenclature of PCV3 genotypes by specialist groups.

PCV3 has been reported to be related with PDNS, reproductive disorders, congenital tremors, and multi-systemic inflammations in pigs [4, 5, 19]. Recent studies showed that there were a high detection rate of PCV3 in weaned pig with severe respiratory disease or diarrhea disease and the PCV3 strains are associated with both digestive and respiratory diseases in swine [6, 20]. However, there was also report which indicated that the positive PCV3 rate in healthy pigs was higher (29.8%) than that in unhealthy pigs (17.9%) [21]. Combined with our research, it suggested that the pigs could live with PCV3 infection without any clinical signs. Thus, The PCV3 maybe as a co-pathogenic factor with other pathogens such as PCV2, porcine epidemic diarrhoea virus (PEDV) and porcine reproductive and respiratory syndrome virus (PRRSV). It is imperative to isolate PCV3 on cell lines and further investigations into the pathogenesis are needed to ascertain the role of PCV3 in swine health [13, 22].

## Conclusions

In conclusion, our study suggested that the oral fluids could be useful to monitor the prevalent status of PCV3 in swine herds due to broad sampling ranges. PCV3 is widely circulating in pigs in Henan province, central China. Subgroup specific amino acids (V27A, K27R, S77 T and I150L) in cap protein should be helpful for further clustering of PCV3. Our study enriched the genetic information and provided new insights into the molecular characteristics of PCV3.

## Methods

### Sample collection and pre-treatment

A total of 318 oral fluid samples (pen-based consist of 20~ 25 pigs and stall-based for individual sow) were collected from five large-scale pig farms in Henan, China. All the pig farms were commercial farrow-to-finisher farms with 300 to 1000 sows and carried out batch production system. The sampled pigs were clinically healthy with a good production performance and normal feed intake. No obvious clinical symptoms, including returns to oestrus or abortion for sows and cough, panting or diarrhea for fatten pigs, were observed. Oral fluids were collected as previous study [13]. Briefly, a 1.2 m length and 1.5 cm diameter cotton rope was suspended within the pen or stall. Pigs chewed on the rope for 30~40 min until drenched enough. The oral fluids were then obtained by squeezing the moistened rope in a plastic bag and cutting one corner of the bag to drain the fluid into a 2 mL EP tubes. Then samples were centrifuged at 12,000 rpm for 2 min to separate any debris. All supernatants were aliquoted and stored at − 80 °C for further studies.

### DNA extraction and PCR

Viral DNA was extracted from each sample using TaKaRa MiniBEST Viral RNA/DNA Extraction Kit Ver.5.0 according to the manufacturer’s instructions. The primers used in this study were listed in Table 3. Briefly, a pair of primer to generate a 330-bp products was used to detect the PCV3 cap gene from each sample as previously described [5]. Five pairs of primers were used for the complete genome and cap gene sequencing by a nested PCR method. The detection primers of PCV2 were designed according to the cap genes. For the PCR reaction, PrimeSTAR Max DNA Polymerase was purchased from TaKaRa and the reaction conditions were as follows: one cycle at 98 °C for 2 min; 30 cycles at 98 °C for 10 s, 55 °C for 15 s and 72 °C for 30 s or 45 s according to the products length, followed by elongation at 72 °C for 5 min and hold at 16 °C. The PCR products were visualized by 1.2% agarose gel electrophoresis and ultraviolet light.

**Table 3:** Primers used in this study

Primer name	Nucleotide Sequence	Primer location (nt)	Product length (bp)	Purpose	Resource
PCV3-D-F	CCACAGAAGGCGCTATGTC	1909–1927 ^a^	330	Detection	[4]
PCV3-D-R	CCGCATAAGGGTCGTCTTG	1599–1617 ^a^			
PCV3-N2-F	AGGAGGTTCACTAAGGTTGT	1026–1045 ^a^	1313	Genome sequencing (nested PCR For G2 fragment)	This study
PCV3-N2-R	CTCTTTGCCGATAATAAGGTAT	317–338 ^a^			
PCV3-G2-F	TTGCACTTGTGTACAATTATTGCG	1113–1136 ^a^	1075		[3]
PCV3-G2-R	ATCTTCAGGACACTCGTAGCACCAC	2163–0-187 ^a^			
PCV3-G1-F	TAGTATTACCCGGCACCTCGGAACC	1994-0-18 ^a^	1257	Genome sequencing (nested PCR For N1 fragment)	[3]
PCV3-G1-R	ACAGGTAAACGCCCTCGCATGTGGG	1226–1250 ^a^			
PCV3-N1-F	GATGAAGCGGCCTCGTGT	106–123 ^a^	1064		This study
PCV3-N1-R	CACCCACCCTCCCAATAA	1152–1169 ^a^			
PCV3-Cap-F	ATGAGACACAGAGCTATATT	1961–1980 ^a^	645	Cap gene sequencing	This study
PCV3-Cap-R	TTAGAGAACGGACTTGTAAC	1336–1355 ^a^			
PCV2-F	AAGGGCTGGGTTATGGTATG	1621–1640 ^b^	353	Detection	This study
PCV2-R	CGCTGGAGAAGGAAAAATGG	187–206 ^b^			

Numbers correspond to positions within the PCV3 strain KY418606 ^a^ and PCV2 strain AF055394 ^b^

### Cloning and sequencing

The PCR products were purified by E.Z.N.A.® Gel Extraction Kit (Omega Bio-tek, Inc.) and then cloned into pMD20-T vector (TaKaRa Biotechnology Co., Ltd., Dalian, China). At least three positive clones were submitted to Sangon Biotech (Shanghai, China) for sequencing.

### Homological and phylogenetic analysis

The 55 complete genome sequences of PCV3 available in NCBI and some reference strains of PCV1 and PCV2 were downloaded for further analysis (Additional file 1: Table S1). Nucleotide alignment and deduced amino acid (aa) sequences (for cap gene) were performed by clustal W method using the DNASTAR software. The phylogenetic tree was constructed by using the neighbor-joining method with the Kimura 2-parameter model in MEGA6.0 software with bootstrap analysis of 1000 replicates [23] .

## Additional file

Additional file 1: **Table S1.** Sequence information in this study. (DOC 89 kb)

## Data Availability

The datasets used and/or analyzed during the current study are available from the corresponding author on reasonable request.

## Abbreviations

A: Alanine
AA: Amino acid
Cap: Capsid protein
I: Isoleucine
K: Lysine
L: Leucine
Nt: Nucleotide
PCV: Porcine circovirus
PCVAD: PCV2-associated disease
PDNS: Porcine dermatitis and nephropathy syndrome
PEDV: Porcine epidemic diarrhoea virus
PMWS: Post-weaning multi-systemic wasting syndrome
PRRSV: Porcine reproductive and respiratory syndrome virus
R: Arginine
S: Serine
ssDNA: Single-stranded DNA
T: Threonine
V: Valine
