# Supplementary material for: Detection and genetic characteristics of porcine circovirus 3 based on oral fluids from asymptomatic pigs in central China
Source: BMC Vet Res. 2019 Jun 13;15:200. doi: 10.1186/s12917-019-1952-3 (PMC6567530; doi:10.1186/s12917-019-1952-3)
Supplement: Supplementary file 1 — Table S1. Sequence information in this study. (DOC 89 kb) [file 12917_2019_1952_MOESM1_ESM.doc]

Additional file 1: Table S1: Sequence information in this study.

| No. | Strain name | Accession No. | Origin | No. | Strain name | Accession No. | Origin |
| --- | --- | --- | --- | --- | --- | --- | --- |
| 1 | PCV3-US/SD2016 | KX966193 | USA, 2016 | 22 | PCV3/CN/GDQG1/2017 | MF405275 | China, 2017 |
| 2 | PCV3-US/MN2016 | KX898030 | USA, 2016 | 23 | PCV3/CN/GDSJ1/2017 | MF405271 | China, 2017 |
| 3 | PCV3-US/MO2015 | KX778720 | USA, 2015 | 24 | PCV3/CN/Jiangxi-B1/2017 | MF589107 | China, 2017 |
| 4 | PCV3-IT/MN2017 | MF162299 | Italy, 2017 | 25 | PCV3/CN/Jiangxi-3/2016 | MF589106 | China, 2016 |
| 5 | PCV3-IT/CO2017 | MF162298 | Italy, 2017 | 26 | PCV3/CN/Guangdong-SG1/2016 | MF589105 | China, 2016 |
| 6 | PCV3-BR/RS/8 | MF079254 | Brazil, 2016 | 27 | PCV3/CN/Guangdong-MX3/2015 | MF589104 | China, 2015 |
| 7 | PCV3-BR/RS/6 | MF079253 | Brazil, 2016 | 28 | PCV3/CN/Guangdong-HZ4/2015 | MF589103 | China, 2015 |
| 8 | PCV3/Thailand/PB01/17 | MG310152 | Thailand, 2017 | 29 | PCV3/CN/Guangdong-HY1/2016 | MF589102 | China, 2016 |
| 9 | PCV3/KU-1609 | KY996345 | South Korea, 2016 | 30 | PCV3/CN/Anhui-14/201611 | MF084994 | China, 2016 |
| 10 | PCV3/KU-1608 | KY996344 | South Korea, 2016 | 31 | PCV3/CN/Shandong-2/201703 | KY778777 | China, 2017 |
| 11 | PCV3/KU-1607 | KY996343 | South Korea, 2016 | 32 | PCV3/CN/Shandong-1/201703 | KY778776 | China, 2017 |
| 12 | PCV3/KU-1606 | KY996342 | South Korea, 2016 | 33 | PCV3-China/GD2016 | KY418606 | China, 2016 |
| 13 | PCV3/KU-1605 | KY996341 | South Korea, 2016 | 34 | PCV3/CN/GDHE2/2016 | MF069116 | China, 2016 |
| 14 | PCV3/KU-1604 | KY996340 | South Korea, 2016 | 35 | PCV3/CN/GDLC1/2016 | MF069115 | China, 2016 |
| 15 | PCV3/KU-1603 | KY996339 | South Korea, 2016 | 36 | PCV3/CN/GXHJ2/2017 | MF405277 | China, 2017 |
| 16 | PCK3-1701 | MF611876 | South Korea, 2016 | 37 | PCV3/CN/GXLJ1/2017 | MF405276 | China, 2017 |
| 17 | PCV3/KU-1602 | KY996338 | South Korea, 2016 | 38 | PCV3/CN/GXLJ2/2017 | MF405274 | China, 2017 |
| 18 | PCV3/KU-1601 | KY996337 | South Korea, 2016 | 39 | PCV3/CN/GXHJ1/2017 | MF405273 | China, 2017 |
| 19 | PCK3-1703 | MF611878 | South Korea, 2016 | 40 | PCV3/CN/GDBL1/2017 | MF405272 | China, 2017 |
| 20 | PCK3-1702 | MF611877 | South Korea, 2016 | 41 | PCV3-China/GX2016-3 | MF155643 | China, 2016 |
| 21 | PCV3-Chian/GX2016-2 | MF155642 | China, 2016 | 42 | DK1980PMWSfree | EU148503 | Denmark, 2008 |
| 43 | PCV3-China/GX2016-1 | MF155641 | China, 2016 | 65 | HeNYC-2, complete genome | MH184537 | China, Henan, 2017 |
| 44 | PCV3-CHN/CC2016 | KY421348 | China, 2016 | 66 | HeNYC-3, complete genome | MH184538 | China, Henan, 2017 |
| 45 | PCV3-CHN/GD2016 | KY421347 | China, 2016 | 67 | HeNYS-1, complete genome | MH184539 | China, Henan, 2017 |
| 46 | PCV3/CN/Jiangxi-62/2016 | KY075989 | China, 2016 | 68 | HeNYS-2, complete genome | MH184540 | China, Henan, 2017 |
| 47 | PCV3/CN/Chongqing-156/2016 | KY075994 | China, 2016 | 69 | HeNYS-3, complete genome | MH184541 | China, Henan, 2017 |
| 48 | PCV3/CN/Chongqing-155/2016 | KY075993 | China, 2016 | 70 | HeNYS-4, complete genome | MH184542 | China, Henan, 2017 |
| 49 | PCV3/CN/Chongqing-150/2016 | KY075992 | China, 2016 | 71 | HeNMJ-1, complete genome | MH184535 | China, Henan, 2017 |
| 50 | PCV3/CN/Chongqing-148/2016 | KY075991 | China, 2016 | 72 | HeNLY-1, complete genome | MH184533 | China, Henan, 2017 |
| 51 | PCV3/CN/Chongqing-147/2016 | KY075990 | China, 2016 | 73 | HeNLY-2, complete genome | MH184534 | China, Henan, 2017 |
| 52 | CHN_Shanghai_0708_2016 | KY865243 | China, 2016 | 74 | HeNYC-4, cap gene | MH184555 | China, Henan, 2017 |
| 53 | CHN_Shanghai_0706_2016 | KY865242 | China, 2016 | 75 | HeNYC-5, cap gene | MH184556 | China, Henan, 2017 |
| 54 | PCV3/CN/Henan-13/2016 | KY075988 | China, 2016 | 76 | HeNYC-6, cap gene | MH184557 | China, Henan, 2017 |
| 55 | PCV3/CN/Fujian-12/2016 | KY075987 | China, 2016 | 77 | HeNYC-7, cap gene | MH184558 | China, Henan, 2017 |
| 56 | PCV3/CN/Fujian-5/2016 | KY075986 | China, 2016 | 78 | HeNYC-8, cap gene | MH184559 | China, Henan, 2017 |
| 57 | Fh17 (PCV2) | AY322004 | France, 2003 | 79 | HeNYC-9, cap gene | MH184560 | China, Henan, 2017 |
| 58 | SZ (PCV2) | AY181948 | China, 2002 | 80 | HeNYC-10, cap gene | MH184561 | China, Henan, 2017 |
| 59 | HBxz-PCV2a | FJ870968 | China, 2008 | 81 | HeNYC-11, cap gene | MH184562 | China, Henan, 2017 |
| 60 | PCV1-Eng-1970 | KJ408798 | United Kingdom, 1990 | 82 | HeNYC-12, cap gene | MH184563 | China, Henan, 2017 |
| 61 | PK (PCV1) | DQ650650 | China, 2006 | 83 | HeNYS-5, cap gene | MH184564 | China, Henan, 2017 |
| 62 | Porcine circovirus 2 | AF055392 | Canada, 1998 | 84 | HeNYS-6, cap gene | MH184565 | China, Henan, 2017 |
| 63 | Porcine circovirus 2 | AF055394 | France, 1998 | 85 | HeNYS-7, cap gene | MH184566 | China, Henan, 2017 |
| 64 | HeNYC-1, complete genome | MH184536 | China, Henan, 2017 | 86 | HeNMJ-2, cap gene | MH184551 | China, Henan, 2017 |
| 87 | HeNMJ-3, cap gene | MH184552 | China, Henan, 2017 | 93 | HeNLY-6, cap gene | MH184546 | China, Henan, 2017 |
| 88 | HeNMJ-4, cap gene | MH184553 | China, Henan, 2017 | 94 | HeNLY-7, cap gene | MH184547 | China, Henan, 2017 |
| 89 | HeNMJ-5, cap gene | MH184554 | China, Henan, 2017 | 95 | HeNLY-8, cap gene | MH184548 | China, Henan, 2017 |
| 90 | HeNLY-3, cap gene | MH184543 | China, Henan, 2017 | 96 | HeNLY-9, cap gene | MH184549 | China, Henan, 2017 |
| 91 | HeNLY-4, cap gene | MH184544 | China, Henan, 2017 | 97 | HeNLY-10, cap gene | MH184550 | China, Henan, 2017 |
| 92 | HeNLY-5, cap gene | MH184545 | China, Henan, 2017 |  |  |  |  |
